# Steller Sex: Infidelity and Sexual Selection in a Social Corvid (*Cyanocitta stelleri*)

**DOI:** 10.1371/journal.pone.0105257

**Published:** 2014-08-22

**Authors:** Katlin R. Overeem, Pia O. Gabriel, Jeff A. Zirpoli, Jeffrey M. Black

**Affiliations:** Department of Wildlife, Humboldt State University, Arcata, California, United States of America; CNRS, France

## Abstract

Genetic analysis of avian mating systems has revealed that more than 70% of monogamous species show incidence of offspring parentage that does not match the social partner. Extra-pair parentage (EPP) has been linked to a variety of factors, including size and symmetry of ornamental traits, coloration, resource availability, and local conspecific density. We examined how ornamental plumage traits of individual Steller's jays (*Cyanocitta stelleri*) and territory characteristics influence genetic fidelity of socially monogamous pairs. We used seven highly polymorphic microsatellite markers to assign paternity to 79 offspring, and identified 12 (15.2%) as extra-pair young (EPY). Steller's jays with extra-pair young had significantly lower values of feather brightness and hue, indicating more ultraviolet-blue shifted coloration, and nested in closer proximity to the forest edge than Steller's jays with no detected EPY. Body size, crest height, asymmetry of ornamental crest stripes, as well as vegetative composition of territories and their proximity to supplemental feeders appeared to have little relationship to EPP. These results indicate that extra-pair parentage plays a role in the evolution of secondary sexual characteristics in both sexes, and suggest local density and availability of resources may influence Steller's jay mating dynamics.

## Introduction

The resource-based nature of serial monogamy makes it the most prevalent mating strategy in avian species with finite resource availability [1], [2]. However, genetic analysis of these systems has revealed more than 70% of monogamous species to have nests containing young with parentage matching the social mother, though not the social father [3]–[5]. Rates of extra-pair copulations (EPC) and resulting extra-pair parentage (EPP) vary between species and populations [6]. Griffith et al. [5] suggest that the considerable variation in EPP among species could be explained by differences in life history and social structure, while within species EPP may vary according to ecological restrictions.

The discrepancy in energy demands between the sexes generally allows males to copulate indiscriminately to increase their fitness, while females are restricted by the costs of reproduction and are thus more selective [7], [8]. The ‘constrained female hypothesis’ posits that female engagement in EPCs is further limited by social and ecological constraints, particularly with respect to the need for paternal assistance [9]. In many species, female participation in EPCs has been associated with decreased paternal care and food provisioning by male pair bond partners, which can be energetically costly to females when resources are not plentiful [9]–[12], but see [13]. In spite of retaliation by social partners, females often continue to seek EPCs, suggesting EPP confers advantages [14].

Several hypotheses attempt to explain the pervasiveness of EPC in socially monogamous birds. From the female's perspective, mixed paternity broods from EPCs may function to increase genetic diversity of a brood, thereby increasing the probability that at least some offspring will survive, which would be advantageous in dynamic and spatially heterogeneous environments [15], [16]. The good genes hypothesis suggests females engage in EPC with males of higher genetic quality than their social mate to increase the genetic quality of her offspring [17]. Sexual selection theory posits that elaborate ornamental traits evolved as honest indicators of quality, as they are energetically costly to produce and maintain [18]–[21]. Moreover, the degree of symmetry in bilateral traits provides insight into developmental stability, as this capability to allocate energy to the production of complex traits allows dependable assessment of individual quality [22].

Studies have linked paternity success to a variety of ornamental traits, including length and symmetry of crest [23] and tail feathers [24]–[26], and intensity of ultraviolet (UV) coloration [27]–[29]. Other studies have established positive within-species relationships between local conspecific density and rates of EPP [30]–[32]. Variation in population density is often associated with habitat characteristics and food availability [33]. For example, Steller's jays (*Cyanocitta stelleri*), a generalist corvid, exist in higher densities along forest edges [34] and are attracted to areas with readily available anthropogenic food sources [35], [36]. Most corvids have been described as unusual because they have very little or no occurrences of EPP [37]–[40]. However, Steller's jays nesting in more crowded areas may encounter conspecifics more frequently, and consequently be provided with increased opportunity for EPCs. Based on the hypotheses outlined above, we examined how ornamental plumage traits and ecological characteristics of Steller's jay territories influence genetic fidelity of socially monogamous pairs.

## Materials and Methods

### Study Species

Steller's jays are a long-lived species, forming long-term pairs that work together to raise young and defend year-round territories [41]–[43]. Sexes are apparently monomorphic, boldly colored with structurally based blue plumage that reflects light in the UV spectrum (Figure S1), contrasted by a dark hood and exaggerated, expressive crest with paired blue vertical stripes [44], [45].

### Study Area

We examined a suburban population of individually marked Steller's jays that has been intensively monitored since 2005 in Arcata, California (40^o^59′N, 124^o^06′W). This population lives year-round on the fringe of second growth redwood (*Sequoia sempervirens*) forest. Steller's jays in this area utilize feeding stations modified with sliding doors that can be drawn closed to selectively capture individuals [46]. These stations are spaced throughout the study area and periodically stocked with peanuts to aid in the observation and selective capture of jays. All work was approved by Humboldt State University's Institutional Animal Care and Use Committee Protocol (Capture and banding: # 08/09.W.14.A; Feather analysis: # 08/09.W.36.A; Genetics: # 10/11.W.76.E). Data was collected as quickly and efficiently as possible to minimize stress to the animal.

### Banding and Physical Measurements

We used 400 µl blood samples, extracted from the brachial vein of Steller's jays during annual capture and banding procedures from 2006–2008. These samples were preserved in Lithium-Heparin, and frozen until molecular analyses. Gender was routinely recorded using sex-specific calls during field observations [41], [47], and confirmed via genetic sexing (unpublished data). Body size (wing chord, gape and tarsus lengths), crest height, and symmetry of eye stripes were routinely recorded during capture events (see [42]), and secondary seven (S7) feathers pulled for plumage color measurements. Zirpoli [45] measured patches on these feathers for three descriptors of coloration: chromatic variables hue (color, indicated by peak reflectance wave lengths) and UV chroma (saturation of UV color), as well as the achromatic, brightness (amount of light reflected relative to a white standard), following the methods of Montgomerie [48]. These values were restricted to wavelengths between 300 and 700 nm and quantified using an Ocean Optics S2000 spectrometer, a PX-2 xenon pulse lamp and a fiber-optic probe held 90° to the feather (Ocean Optics, Dunedin, Florida, USA) [49], [50]. Original reflectance spectra were extracted using CLR software (1.05) to obtain the three descriptors of coloration [51]. Six measurements were taken from each feather and averaged to calculate final color variables for each individual (for detailed methods see [45]).

Pair status and territory locations were determined by monitoring jays for behavioral data, nesting locations, and breeding status on a near daily basis (see [42], [43], [46]). Pair bonds were inferred through frequent, non-aggressive behavioral interactions, courtship displays, and cooperative manufacture and defense of nests and the surrounding territory during the breeding season (March - August). Although many nest locations were known, the sensitivity of Steller's jays to nest disturbance prohibited us from sampling offspring directly from the nest. Therefore, all young were captured within two months of fledging, prior to dispersal. As a result, we have no evidence of differential chick mortality prior to fledging.

### Habitat Measurements

To define territories we used ArcGIS 10.0 (ESRI 2011) to create 100 m radius buffers around previously identified nest locations (recorded in NAD83 UTM Zone 16N using a Garmin GPS). These territory sizes were chosen to reflect core use areas of similar size of those measured using radio telemetry with Steller's jays (WP Goldenberg, personal communication). To avoid pseudoreplication for pairs with multiple nest attempts in the same year (10% of cases), we found the centroid point among nests around which to place the territory region. We quantified habitat characteristics within these territories by digitizing polygons from BING base map imagery (resolution to 0.5 m, 2010), available for use in ArcMap 10.0.

We measured percent cover within regions for the categories 1) conifers 2) hardwoods, 3) shrubs, 4) grass and dirt plots and 5) impervious surface (following [36]). Structures, paved areas, and vegetation classifications difficult to discern in ArcGIS were verified in person. We used model builder in ArcCatalog to determine percent cover for every classification type within each region and measured distances from nests to nearest creek, forest boundary, and baited feeder traps. We treated territories for a pair associated with assigned young independently to allow for changes in location and attributes across years, as well as any switch in mates. In the incidence when no nests were located for a pair in a given year in which young were attributed to them (*n* = 7), we used the most recently gathered habitat data for that pair, as territories did not differ substantially between years. Finally, we depicted the distribution of territories on a map to allow for visual interpretation.

### Microsatellite Genotyping

To prepare blood samples for analysis, we used a traditional phenol-chloroform method for DNA extraction with proteinase K digestion [52]. We preserved the purified DNA in TE buffer (10 mM Tris, HCl, 1 mM EDTA, pH 8.0). DNA amplification via polymerase chain reaction (PCR) was carried out in a Thermal Cycler (Thermo Cycler 2720, Applied Biosystems, Foster City, CA). To assign parentage we used seven highly polymorphic microsatellite markers, six developed for the Florida Scrub-jay (*Aphelocoma coerulescens*): Apco2, Apco29, Apco30, Apco37, Apco40, Apco41 [53], [54], and one developed for the Mexican jay (*Aphelocoma ultramarina*): MJG3 [55]. We ran PCR amplification with complementary forward and reverse primers with M13 tails and products were separated on a 2.5% polyacrylamide gel and imaged by a Li-COR DNA 4300 Analyzer Gene Readir (LI-COR Biosciences, Lincoln, NE). All gels included negative controls (PCR blanks) in order to assess possible contamination. Workplaces and instruments were thoroughly sterilized prior to molecular work. All genetic analyses were conducted at the Biology Core Facility at Humboldt State University in Arcata, CA. This facility has a divided laboratory to keep high-copy samples separate from low-copy samples in order to reduce contamination.

### Parentage Analysis

We assigned both maternal and paternal parentage to offspring using CERVUS 3.2 [56], separating the analyses by birth year to avoid the possibility of an individual's offspring being mistakenly assigned as a candidate parent. To assess confidence in parent assignments, we used Delta-scores, the difference between the logarithms of the likelihood ratios (LOD) of the first and second most likely candidate parents. We ran separate simulations for each year in CERVUS to account for yearly variation in the number of known candidate parents, which allows for different critical Delta values each year. We set the proportion of the population sampled to calculated average annual recapture estimates for this population (ρ = 0.79, unpublished data), as done in similar study on Siberian jays [39]. We conservatively set genotyping error to 1%, as most samples were genotyped and checked at least twice. Bands were measured automatically using GeneProfiler 4.05 (Scanalytics, Inc., Rockville, MD) and corrected manually.

We included all adult birds that were possibly alive in a given birth year as parent candidates in the analysis; however, individuals hatched the prior year (second years) were included only if their presence was noted in the months preceding a given breeding season. We excluded all unobserved second year birds due to high dispersal rate of juveniles and to minimize the possibility of a non-present individual being assigned as a parent to a younger sibling. All individuals genotyped at fewer than five microsatellite loci were excluded from analysis.

Within-pair young (WPY) were assigned when the most likely candidate parents given by CERVUS 3.2 for an offspring matched a known social pair. In a few instances, a known social pair was given as the second or third most likely parent pair. In all of these cases the Delta scores between top putative parents were small and no candidate set could be identified with 95% confidence, therefore we assumed that these offspring belonged to the known social pair. Possible EPPs were considered only when no known social pairs had positive LOD scores, and one or both candidate parents could be assigned with 95% confidence.

### Statistical Analysis

We calculated the mean of repeated measurements for physical variables of individuals, including only those measured on adult birds prior to the end of the time frame of this study, for use in analysis. Because of the apparent monomorphism of Steller's jays, we analyzed both sexes together and treated extra-pair parentage as a binomial response variable (EPP = 1, No EPP = 0). We separately examined the influence of physical traits and nesting habitat characteristics using logistic regression models that were pre-selected based on relevant hypotheses. Regression models for appearance and habitat were ranked separately according to Akiake's Information Criterion (AIC) and adjusted for small sample size (AICc). Relative strengths of top models were assessed using evidence ratios [57].

To reduce the number of tested parameters, we performed Principal Component Analyses (PCA) to create an index of body size using wing chord, gape and tarsus lengths [58]. The composite size variable PC1 explained 81% of the variance. In order to allow better interpretability we chose not to PCA color variables [59]. We included sex as a parameter in models that contained variables known to be dimorphic in Steller's jays (unpublished data).

In Steller's jay territories, overall vegetative cover (sum of conifers, hardwoods, and shrubs) was correlated with the proportion of impervious surface (Spearman's rank correlation: r_s_ = −0.894, *n* = 39, *P*<0.001), therefore we only included the latter in the analysis in order to reduce the number of candidate models. Additionally, conifer cover and distance from edge were correlated (r_s_ = −0.561, *n* = 39, *P*<0.001), so we included only distance from edge as an analysis variable. Due to missing or incomplete data, 20 individuals were eliminated from the appearance analysis and 8 nesting pairs were eliminated from the territory analysis. We chose not to model average competitive models because of the explorative rather than predictive nature of this study [57], [60]. All statistical analyses were conducted in Program R 2.12 (R Development Core Team 2011).

## Results

We analyzed blood sampled from 242 Steller's jays and genotyped 99.6% of the 242 at five microsatellite loci or more, allowing parental assignment to a total of 79 offspring over all three years (Table 1, 2). We assigned 67 WPY to a total of 25 known social pairs, and identified 12 (15.2%) offspring as extra-pair young (EPY). In these cases, a member of a known social pair was identified at the 95% confidence interval as one parent, and the known social mate was eliminated as a possible genetic contributor to that offspring. Low Delta scores prevented confident assignment of the extra-pair mate for 75% of these cases, thus we restricted our statistical analysis to the 11 parents that gained EPY (6 males, 5 females) identified at the 95% confidence interval (Table 2). In most cases, only one extra-pair young was detected per individual, with the exception of one male with two extra-pair young detected in the same year and one female with one extra-pair young detected in two different years. All parents with EPY were three years old or greater, as were their cuckolded mates. Of the parents with WPY only, 88.6% were also three years old or greater, while the remaining eight were successful second year breeders.

**Table 1:** Microsatellites used in genetic parentage analysis of Steller's jays.

Locus	A	n	H_O_	H_E_	PIC	N-Excl_1_	N-Excl_2_	N-Excl_pair_	N-Excl_id_
Apco2	18	238	0.924	0.903	0.893	0.333	0.200	0.064	0.018
Apco29	9	242	0.769	0.745	0.713	0.643	0.460	0.264	0.097
Apco30	16	242	0.938	0.911	0.902	0.312	0.184	0.055	0.015
Apco37	17	240	0.921	0.871	0.857	0.409	0.256	0.096	0.029
Apco40	11	238	0.824	0.838	0.816	0.496	0.326	0.153	0.047
Apco41	8	241	0.780	0.725	0.685	0.674	0.496	0.303	0.115
MJG3	28	220	0.932	0.924	0.916	0.272	0.158	0.041	0.011
Combined	-	-	0.870	0.845	0.826	0.002	∼0.00	∼0.00	∼0.00

Number of alleles (A), individuals genotyped (n), observed (H_o_) and expected (H_E_) heterozygosity, non-exclusion probability for first (N-Excl_1_) parent, second (N-Excl_2_) parent when first is assigned, parent pair (N-Excl_pair_), and individual identity (N-Excl_id_). Non-exclusion probability refers to the probability that the true parent will be falsely excluded as a candidate parent.

**Table 2:** Genetic assignment of parents and offspring where within-pair young (WPY) are the genetic offspring of a social pair and extra-pair young (EPY) are not.

	Parents			Offspring	
Year	Pairs: WPY only	Females with EPY	Males with EPY	WPY	EPY
2006	9	1	1	16	2
2007	12	2	2	25	5
2008	14	3	3	26	5

AIC ranking of logistic regression models of appearance revealed two competitive models predicting extra-pair parentage, the top ranked model including brightness and sex (weight 0.51), and the second with hue (weight 0.33, Table 3). Evidence ratios between these competitive models show the best model to have 1.5 times the weight of evidence relative to the second best model. Compared to the null model, the best model including brightness and sex, and the second best model including hue, had 8.9 and 5.7 times the weight of evidence, respectively. The coefficients of these top models indicate that Steller's jays with extra-pair parentage had lower values of feather brightness and hue, meaning their feathers reflect fewer long wavelengths and have, on average, shorter, more ultraviolet wavelengths than faithful jays (Figure S2; S3). Size composite, crest height, and asymmetry of crest stripes appeared to have little relationship to EPP, as models with these variables had AIC weights less than 0.06 (Table 3).

**Table 3:** Logistic regression models describing extra-pair paternity (yes/no) and physical characteristics of Stellers jays.

Model	K	Log Likelihood	AICc	ΔAICc	Akaike Weight	Cumulative Weight
BRIGHTNESS + SEX	3	−15.323	37.331	0.000	0.507	0.507
HUE	2	−16.937	38.207	0.876	0.327	0.834
CHROMA + SEX	1	−19.790	41.463	4.131	0.064	0.898
NULL	2	−18.901	41.688	4.356	0.057	0.955
CREST HEIGHT + SEX	3	−18.567	43.820	6.489	0.020	0.975
SIZE + SEX	2	−19.783	43.900	6.568	0.019	0.994
ASYM OF CREST STRIPES	3	−19.750	46.186	8.855	0.006	1.000

**Figure 1:**
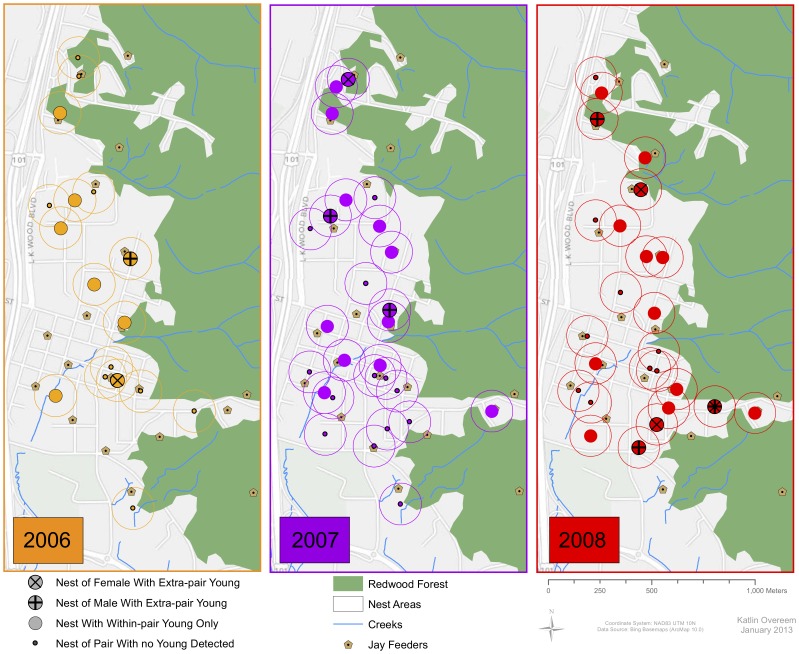
Territories of Steller's jay pairs displayed based on genetic mate fidelity of the territory owners.

Logistic regression models including measures of habitat within Steller's jay pair territories as predictors of extra-pair parentage revealed a notable negative relationship between nesting locations of parents with EPY and proximity to the forest edge when ranked by AIC (Table 4, Figure 1; S4). This model had 2.8 times the weight of evidence relative to the second model, and 37.9 times the weight of evidence relative to the null model. This model received a third of the weight; however, models including distances from the nearest creek and feeder, and proportions of shrub, hardwood and impervious surfaces cover each had AIC weights around 0.12 (Table 4).

**Table 4:** Logistic regression nesting habitat models describing extra-pair paternity (yes/no) of Stellers jays.

Model	K	Log Likelihood	AICc	ΔAICc	Akaike Weight	Cumulative Weight
DISTANCE FROM FOREST EDGE	2	−21.957	48.246	0.000	0.379	0.379
SHRUB	2	−23.000	50.333	2.086	0.133	0.512
DISTANCE FROM FEEDER	2	−23.050	50.433	2.187	0.127	0.639
DISTANCE FROM CREEK	2	−23.062	50.458	2.211	0.125	0.764
IMPERVIOUS SURFACE	2	−23.143	50.620	2.373	0.116	0.880
HARDWOODS	2	−23.188	50.709	2.463	0.111	0.990
NULL	1	−26.701	55.510	7.263	0.010	1.000

## Discussion

Genetic parentage construction revealed moderate rates of EPP in an urban fringe population of Steller's jays in northern California (15% of 79 typed progeny). This is the first study to examine the fidelity of Steller's jays and subsequently report the occurrence of EPP in the species. Assessments of extra-pair behavior in other corvid species report a range of incidence from 0% occurrence for the colonial-nesting Jackdaw (*Corvus monedula*), monogamous Siberian jays (*Perisoreous infaustus*) and cooperative breeding Florida Scrub-jays, to as high as 40% of nestlings assigned to group members other than the cooperative partner (one of the highest rates reported) in the plural breeding Mexican jay [37]–[40], [61]. This within-taxon variation in mating system dynamics (reviewed in [62]) is likely explained through social and ecological distinctions, where resource availability often restricts time-budgets and limits opportunities for extra-pair copulations [5], [12].

The UV reflectance of Steller's jays is a non-iridescent structural coloration, produced through incoherent scattering of light on keratin vacuoles surrounding a core melanin layer that serves as a backdrop to intensify the effect [44], [63]. Reflectance of light in the UV spectrum is thought to be an honest signal of quality, as this type of coloration necessitates adequate intake of nutrients to form the fundamental feather microstructure [64]–[67]. Steller's jay courtship occurs long after molt [42], [68]. This may allow individuals to more dependably assess condition of a potential mate, as the feather's resistance to wear is presumably an indicator of structural integrity, serving as an honest signal of the quality of the beholder [19], [69].

We found that the feathers of extra-pair parents of both genders had shorter wavelengths at peak reflectance (i.e. lower hue values indicating color shifted further into UV spectrum) than the feathers of individuals with no extra-pair success. Extra-pair parents were also less bright, which may allow a greater proportion of reflectance in the UV range, and signal more durable, higher quality feathers [45], [50], [70]. Combined, lower values of hue and brightness in Steller's jays translate to more vibrant ultraviolet coloration in individuals with extra-pair young. The evolution of similar ornamentation in males and females may result from mutual mate choice in socially monogamous species [71], [72], particularly when the levels of care are similar and the ornamentation is representative of access to resources important to reproduction [73], [74].

The good genes hypothesis posits that higher quality males will gain superior access to extra-pair copulation opportunities [17]. These results agree with findings of other studies relating UV vibrancy to paternity gains [28], [75], lending support to the good genes hypothesis. For females, higher quality plumage may be selected for more indirectly. The ‘constrained female hypothesis’ predicts that females with higher quality territories have greater access to resources and can therefore afford to risk loss of paternal care as a consequence of EPCs [9]. Since UV ornamentation is indicative of individual quality, we conclude that the more vibrant breeding females with EPY had greater access to resources, freeing them from the constraints of paternal assistance. Our results therefore add to the suggestion that EPCs may facilitate the directional evolution of sexually selected ornamentation species where the social mating systems limit this opportunity.

Steller's jays with EPY nested in closer proximity to the forest than those with WPY only (Figure 1), which could be related to inflated densities reported at these locations [34], since denser populations may allow more opportunity for EPCs. Additionally, inflated densities at forest edges may be indicative of higher quality habitat [33], further supporting the notion that females occupying higher quality territories can afford to engage in EPCs. Studies on Steller's jay preference for forest edges are lacking, though jays are commonly observed utilizing the tops of trees to view the landscape and call long distances, and may use forest edges as travel and dispersal corridors. In these types of forest canopies, ambient light is rich in blue and UV wavelengths; therefore selection on coloration strongly reflecting UV enhances conspicuousness [76], [77]. In fact, UV reflecting feathers are often associated with body parts actively moved or erected in sexual displays (such is the case with the jay's crest stripes, wings and tail), conceivably to enhance detection [78]. Given this, we postulate that increased conspicuousness in forested habitats may facilitate the attraction of extra-pair mates, and thus further selection on these traits.

Limited knowledge of social partnerships and delineation of a sampling area in a contiguous population restricted parentage assignment in this population of Steller's jays. Neighboring or floater males that resided just outside our study area, such as in the un-sampled adjacent forest, likely sired extra-pair young of known nesting females. Similarly, failure to assign maternal or any parentage to an offspring is equally likely a consequence of this ‘open population’, as well as a result of not knowing all social partnerships. In some cases we were unable to assign any parentage to an offspring. Because of the inaccessibility of nests and their sensitivity to disturbance [46], young jays needed to be captured within two months after fledging. Since our post-fledge capture method exploits exploratory behavior, it is likely that in most of these cases the young wandered in from outside the study area where parents were un-sampled. It is possible that the occurrence of nest parasitism by un-sampled individuals may have precluded assignment of ‘dumped’ offspring; however, it is unknown whether this behavior exists in Steller's jays, as determining so would require the sampling of offspring directly from the nest.

In summary, our results support the good genes hypothesis, highlighting the importance of UV coloration in sexual selection on males. Additionally, our results indicate concurrent plumage selection on females, which may be explained as a consequence of predictions from the ‘constrained female hypothesis’. Furthermore, this study suggests that local density may play a role in Steller's jay mating strategies, though more research is needed. Steller's jay affinity for urban/wildland interfaces, and their apparent ecological [79], behavioral [46], and genetic (this study) plasticity may enable them to thrive in the face of urban expansion (see [80]). This study not only provides valuable information on the breeding dynamics of an adaptable species, but also contributes to the greater understanding of the complicated relationship between mating systems and the selection for ornamentation in both sexes.

## Supporting Information

Figure S1 **Secondary feathers of an adult Steller's jay illuminated under florescent black light to show UV coloration.**
(TIFF)

Figure S2 **Feather brightness (% total reflectance, 300–700 nm) subdivided by gender and detection of extra-pair parentage (EPP) in breeding Stellers jays.**
(TIFF)

Figure S3 **Comparison of feather hue of adult Steller's jays with and without extra-pair parentage (EPP).**
(TIFF)

Figure S4 **Comparison of nest distance from forest edge (m) of adult Steller's jays with and without extra-pair parentage (EPP).**
(TIFF)

File S1 Dataset for appearance analysis.
(CSV)

File S2 Dataset for habitat analysis.
(CSV)

## References

[1] Lack D (1968) Ecological adaptations for breeding in birds. London: Chapman and Hall.

[2] Black JM (1996) Introduction: pair bonds and partnerships. In: Black JM, editor. Partnerships in birds: the study of monogamy. Oxford: Oxford University Press. pp.3–20.

[3] Birkhead TR, Møller AP (1992) Sperm competition in birds: evolutionary causes and consequences. San Diego: Academic Press.

[4] Birkhead TR, Møller AP (1995) Extra-pair copulation and extra-pair paternity in birds. Anim Behav 49: 843–848.

[5] Griffith SC, Owens IPF, Thuman KA (2002) Extra pair paternity in birds: a review of interspecific variation and adaptive function. Molec Ecol 11: 2195–2212. 12406233 10.1046/j.1365-294x.2002.01613.x

[6] Petrie M, Kempenaers B (1998) Extra-pair paternity in birds: explaining variation between species and populations. TREE 13: 52–58. 21238200 10.1016/s0169-5347(97)01232-9

[7] Bateman AJ (1948) Intra-sexual selection in Drosophila. Heredity 2: 349–368. 18103134 10.1038/hdy.1948.21

[8] Trivers RL (1972) Parental investment and sexual selection. In: Campbell B, editor. Sexual selection and the descent of man. Chicago: Aldine-Atherton. pp.136–179.

[9] Gowaty PA (1996) Battles of the sexes and origins of monogamy. In: Black JM, editor. Partnerships in birds: the study of monogamy. Oxford: Oxford University Press. pp.21–52.

[10] Birkhead TR, Møller AP (1996) Monogamy and sperm competition in birds. In: Black JM, editor. Partnerships in birds: the study of monogamy. Oxford: Oxford University Press. pp.323–343.

[11] Whittingham LA, Dunn PO (2001) Male parental care and paternity in birds. Curr Ornithol 16: 257–298.

[12] Arnold KE, Owens IPF (2002) Extra-pair paternity and egg dumping in birds: life history, parental care and the risk of retaliation. Proc R Soc Lond B 269: 1263–1269. 10.1098/rspb.2002.2013 PMC1691019 12065043

[13] Bouwman KM, Lessells CM, Komdeur J (2005) Male reed buntings do not adjust parental effort in relation to extrapair paternity. Behav Eco 16: 499–506.

[14] Fishman MA, Stone L (2002) Fertility assurance through extrapair fertilization, and male parental effort. B Math Biol 64: 809–823. 10.1006/bulm.2002.0301 12216422

[15] Kempenaers B, Verheyen GR, Dhondt AA (1997) Extrapair paternity in the blue tit (*Parus caeruleus*): female choice, male characteristics, and offspring quality. Behav Ecol 8: 481–492.

[16] Petrie M, Doums C, Møller AP (1998) The degree of extra-pair paternity increases with genetic variability. P Natl Acad Sci USA 95: 9290–9395. 10.1073/pnas.95.16.9390 PMC21348 9689090

[17] Møller AP, Alatalo RV (1999) Good-genes effects in sexual selection. Proc R Soc Lond B 266: 85–91.

[18] Darwin C (1871) The descent of man and selection in relation to sex. London: J. Murrey.

[19] Keyser AJ, Hill GE (1999) Condition-dependent variation in the blue-ultraviolet coloration of a structurally based plumage ornament. Proc R Soc Lond B 266: 771–777.

[20] Ferns PN, Hinsely SA (2004) Immaculate tits: head plumage pattern as an indicator of quality in birds. Anim Behav 67: 261–272.

[21] Jawor JM, Gary N, Beall SM, Breitwisch R (2004) Multiple ornaments correlate with aspects of condition and behaviour in female northern cardinals, *Cardinalis cardinalis* . Anim Behav 67: 875–882.

[22] Møller AP, Swaddle JP (1997) Asymmetry, developmental stability, and evolution. Oxford: Oxford University Press.

[23] Jones IL, Hunter FM (1999) Experimental evidence for mutual inter- and intrasexual selection favouring a crested auklet ornament. Anim Behav 57: 521–528. 10196041 10.1006/anbe.1998.1012

[24] Møller AP (1992) Female swallow preference for symmetrical male sexual ornaments. Nature 357: 238–240. 1589021 10.1038/357238a0

[25] Saino N, Primmer CR, Ellegren H, Møller AP (1997) An experimental study of paternity and tail ornamentation in the barn swallow (*Hirundo rustica*). Evolution 51: 562–570. 28565341 10.1111/j.1558-5646.1997.tb02443.x

[26] Møller AP, Saino N, Taramino G, Galeotti P, Ferrario S (1998) Paternity and multiple signaling: effects of a secondary sexual character and song on paternity in the barn swallow. Amer Nat 151: 236–242. 18811354 10.1086/286114

[27] Hunt S, Cuthill IC, Bennett ATD, Griffiths R (1999) Preferences for ultraviolet partners in the blue tit. Anim Behav 58: 809–815. 10512654 10.1006/anbe.1999.1214

[28] Balenger SL, Johnson SL, Masters BS (2009) Sexual selection in a socially monogamous bird: male color predicts paternity success in the mountain bluebird, *Sialia currucoides* . Behav Ecol Sociobiol 63: 403–411.

[29] O'brien EL, Dawson RD (2010) Plumage color and food availability affect male reproductive success in a socially monogamous bird. Behav Ecol 22: 66–72.

[30] Westneat DF, Sherman PW (1997) Density and extra-pair fertilizations in birds: a comparative analysis. Behav Ecol Sociobiol 41: 205–215.

[31] Charmantier A, Perret P (2004) Manipulation of nest-box density affects extra-pair paternity in a population of blue tits (*Parus caeruleus*). Behav Ecol Sociobiol 56: 360–365.

[32] Møller AP, Ninni P (1998) Sperm competition and sexual selection: a meta-analysis of paternity studies in birds. Behav Ecol Sociobiol 43: 345–358.

[33] Newton I (1998) Population limitation in birds. San Diego: Academic Press.

[34] Brand AL, George TL (2001) Response of passerine birds to forest edge in Coast Redwood forest fragments. Auk 118: 678–686.

[35] Marzluff JM, Millspaugh JJ, Hurvitz P, Handcock MS (2004) Relating resources to a probablistic measure of space use: forest fragments and Steller's jays. Ecology 85: 1411–1427.

[36] Kalinowski RS, Johnson MD (2010) Influence of suburban habitat on a wintering bird community in coastal northern California. Condor 112: 274–282.

[37] Quinn JS, Woolfenden GE, Fitzpatrick JW, White BN (1999) Multi-locus DNA fingerprinting supports genetic monogamy in Florida scrub-jays. Behav Ecol Sociobiol 45: 1–10.

[38] Henderson IG, Hart PJB, Burke T (2000) Strict monogamy in a semi-colonial passerine: the jackdaw *Corvus monedula* . J Avian Biol 31: 177–182.

[39] Gienapp P, Merila J (2010) No evidence for extra-pair paternity in Siberian jays. PlosOne 5: 1–6. 10.1371/journal.pone.0012006 PMC2918499 20711255

[40] Townsend AK, Bowman R, Fitzpatrick JW, Dent M, Lovette IJ (2011) Genetic monogamy across variable demographic landscapes in cooperatively breeding Florida scrub-jays. Behav Ecol 22: 464–470.

[41] Greene E, Davidson W, Muehter V (1998) Steller's jay: No. 343. In: Poole A, Grill G, editors. The Birds of North America. Philadelphia: American Ornithologists Union.

[42] Gabriel PO, Black JM (2012a) Reproduction in Steller's jays (*Cyanocitta Stelleri*): Individual characteristics and behavioral strategies. Auk 3: 377–386.

[43] Gabriel PO, Black JM (2012b) Behavioural Syndromes, partner compatibility and reproductive performance in Steller's jays. Ethology 118: 76–86.

[44] Shawkey MD, Hill GE (2006) Significance of a basal melanin layer to production of non-iridescent structural plumage color: evidence from an amelanotic Steller's jay (*Cyanocitta stelleri*). J Exp Biol 209: 1245–1250. 16547296 10.1242/jeb.02115

[45] Zirpoli J, Black JM, Gabriel PO (2013) Parasites and plumage: an experimental field test of parasite-mediated handicap hypothesis. Ethol Ecol Evol 25: 103–116.

[46] Gabriel PO, Black JM (2010) Behavioral syndrome in Steller's jays: the role of time frames in the assessment of behavioral traits. Anim Behav 80: 689–697.

[47] Hope S (1980) Call form in relation to function in the Steller's jay. Amer Nat 116: 788–820.

[48] Montgomerie R (2006) Analyzing colors. In: Hill GE, McGraw KJ, editors. Bird Coloration, vol 1. Cambridge: Harvard University Press. pp.90–147.

[49] Endler JA (1990) On the measurement and classification of colour in studies of animal colour patterns. Biol J Linn Soc 41: 315–352.

[50] Andersson S, Prager M (2006) Quantifying Colors. In: Hill GE, McGraw KJ, editors. Bird coloration, vol. 1. Cambridge: Harvard University Press. pp.41–89.

[51] Montgomerie R (2008) CLR, version 1.05 [Internet]. Kingston: Queen's University. Available: http://post.queensu.ca/~mont/color/

[52] Sambrook J, Fritsch EF, Maniatis T (1989) Molecular cloning: a laboratory manual, 2nd ed. Cold Spring Harbor: Cold Spring Harbor Laboratory Press.

[53] Stenzler LM, Fitzpatrick JW (2002) Isolation of microsatellite loci in the Florida Scrub-Jay *Aphelocoma coerulescens* . Molec Ecol Notes 2: 547–550.

[54] Burg TM, Gaston AJ, Winker K, Friesen VL (2005) Rapid divergence and postglacial colonization in western North American Steller's jays (*Cyanocitta stelleri*). Mol Ecol 14: 3745–3755. 16202093 10.1111/j.1365-294X.2005.02710.x

[55] Li SH, Huang YJ, Brown JL (1997) Isolation of tetranucleotide repeat microsatellites from the Mexican jay. Molec Ecol 6: 499–501. 9161019 10.1046/j.1365-294x.1997.00215.x

[56] Kalinowski ST, Taper ML, Marshall TC (2007) Revising how the computer program CERVUS accommodates genotyping error increases success in paternity assignment. Molec Ecol 16: 1099–1106. 17305863 10.1111/j.1365-294X.2007.03089.x

[57] Burnham KP, Anderson DR (2002) Model selection and multi-model inference: a practical information-theoretic approach. New York: Springer.

[58] LaBarbera M (1989) Analyzing body size as a factor in ecology and evolution. Annu Rev Ecol Syst 20: 97–117.

[59] Armenta JK, Dunn PO, Whittingham LA (2008) Quantifying avian sexual dichromatism: a comparison of methods. Exp Biol 211: 2423–2430. 10.1242/jeb.013094 18626076

[60] Anderson DR (2008) Model based inference in the life sciences: a primer on evidence. New York: Springer Sciences.

[61] Li S, Brown JL (2000) High frequency of extrapair fertilization in a plural breeding bird, the Mexican jay, revealed by DNA microsatellites. Anim Behav 60: 867–877. 11124886 10.1006/anbe.2000.1554

[62] Townsend AK, Clark AB, McGowen KJ, Lovette IJ (2009) Reproductive partitioning and the assumptions of reproductive skew models in the cooperatively breeding American crow. Anim Behav 77: 503–512. 20126287 10.1016/j.anbehav.2008.10.030 PMC2677701

[63] Prum RO (2006) Anatomy, physics and evolution of avian structural colors. In: Hill GE, McGraw KJ, editors. Bird coloration, vol. 1. Cambridge: Harvard University Press. pp.295–352.

[64] Doucet SM, Shawkey MK, Rathburn HL, Mays HL Jr, Montgomerie R (2004) Concordant evolution of plumage colour, feather microstructure and a melanocortin receptor gene between mainland and island populations of a fairy-wren. Proc R Soc Lond B 271: 1663–1670. 10.1098/rspb.2004.2779 PMC1691780 15306285

[65] Siefferman L, Hill GE, Dobson FS (2005) Ornamental plumage coloration and condition are dependent on age in eastern bluebirds *Sialia sialis* . J Avian Biol 36: 428–435.

[66] Siefferman L, Shawkey MD, Bowman R, Woolfenden GE (2008) Juvenile coloration of Florida scrub-jay (*Alphelocoma coerulescens*) is sexually dichromatic and correlated with condition. J Ornithol 149: 357–363.

[67] Griggio M, Zanollo V, Hoi H (2010) UV plumage color is an honest signal of quality in budgerigars. Ecol Res 25: 77–82.

[68] Brown JL (1964) The integration of agonistic behavior in the Steller's jay (*Cyanocitta stelleri*; Gmelin). Berkeley: University of California Press.

[69] Doucet SM (2002) Structural plumage coloration, male body size, and condition in the blue-black grassquit. Condor 104: 30–38.

[70] Örnborg J, Andersson S, Griffith SC, Sheldon BC (2002) Seasonal changes in a ultra-violet structural colour signal in blue tits, *Parus caeruleus* . Biol J Linn Soc 76: 237–245.

[71] Amundsen T (2000) Why are female birds ornamented? Trends Ecol Evol 15: 149–155. 10717684 10.1016/s0169-5347(99)01800-5

[72] Kraaijeveld K, Kraaijeveld-Smit FJL, Komdeur J (2007) The evolution of mutual ornamentation. Animal Behaviour 74: 657–677.

[73] Johnstone RA, Reynolds JD, Deutsch JC (1996) Mutual mate choice and sex differences in choosiness. Evolution 50: 1382–1391. 28565695 10.1111/j.1558-5646.1996.tb03912.x

[74] Clutton-Brock T (2007) Sexual selection in males and females. Science 318: 1882–1885. 18096798 10.1126/science.1133311

[75] Bitton P, O'Brien EL, Dawson RD (2007) Plumage brightness and age predict extrapair fertilization success of male tree swallows, *Tachycineta biocolor* . Anim Behav 74: 1777–1784.

[76] Gomez D, Théry M (2004) Influence of ambient light on the evolution of colour signals: comparative analysis of a Neotropical rainforest bird community. Ecol Lett 7: 279–284.

[77] Théry M (2006) Effects of light environment on color communication. In: Hill GE, McGraw KJ, editors. Bird coloration, vol. 1. Cambridge: Harvard University Press. pp.148–173.

[78] Hausmann F, Arnold KE, Marshall NJ, Owens IP (2003) Ultraviolet signals in birds are special. Proc R Soc Lond B 270: 61–67. 10.1098/rspb.2002.2200 PMC1691211 12590772

[79] Rockwell C, Gabriel PO, Black JM (2012) Bolder, older, and selective: factors of individual-specific foraging behaviors in Steller's jays. Behav Ecol 23: 676–683.

[80] Evans KL, Hatchwell BJ, Parnell M, Gaston KJ (2010) A conceptual framework for the colonization of urban areas: the blackbird *Turdus merula* as a case study. Biol Rev 88: 643–667. 10.1111/j.1469-185X.2010.00121.x 20128785

