# Supplementary figures and images for: Steller Sex: Infidelity and Sexual Selection in a Social Corvid (Cyanocitta stelleri)
Source: PLoS One. 2014 Aug 22;9(8):e105257. doi: 10.1371/journal.pone.0105257 (PMC4141755; doi:10.1371/journal.pone.0105257)

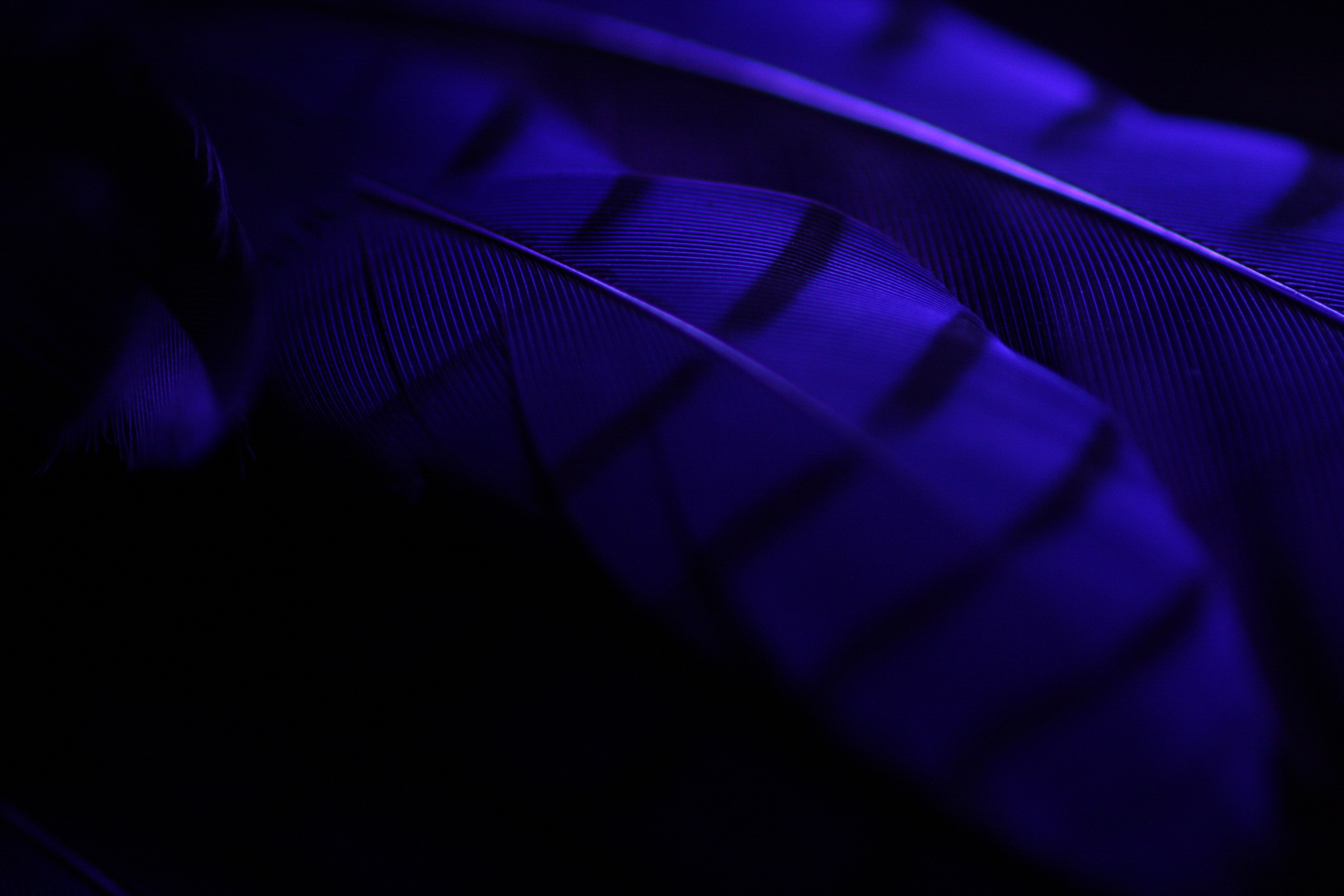

Supplement: Figure S1 — Secondary feathers of an adult Steller's jay illuminated under florescent black light to show UV coloration. (TIFF) [file pone.0105257.s001.tiff]

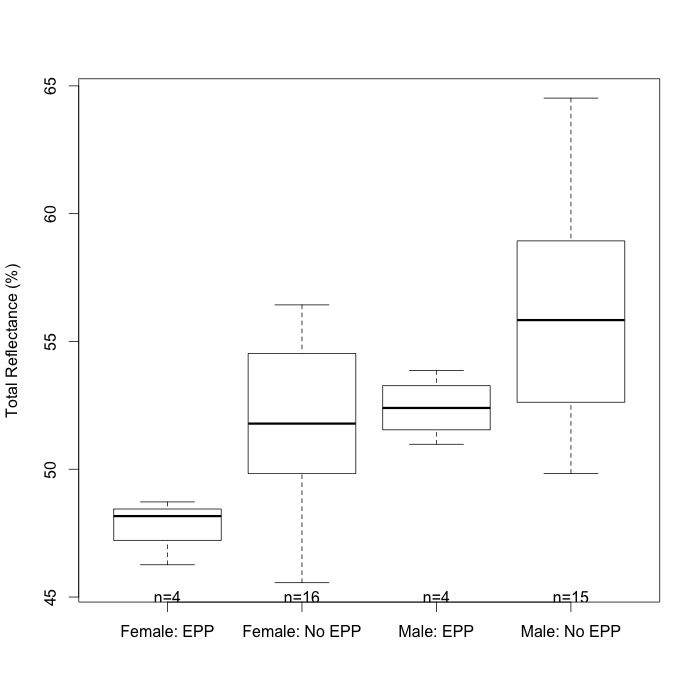

Supplement: Figure S2 — Feather brightness (% total reflectance, 300–700 nm) subdivided by gender and detection of extra-pair parentage (EPP) in breeding Stellers jays. (TIFF) [file pone.0105257.s002.tiff]

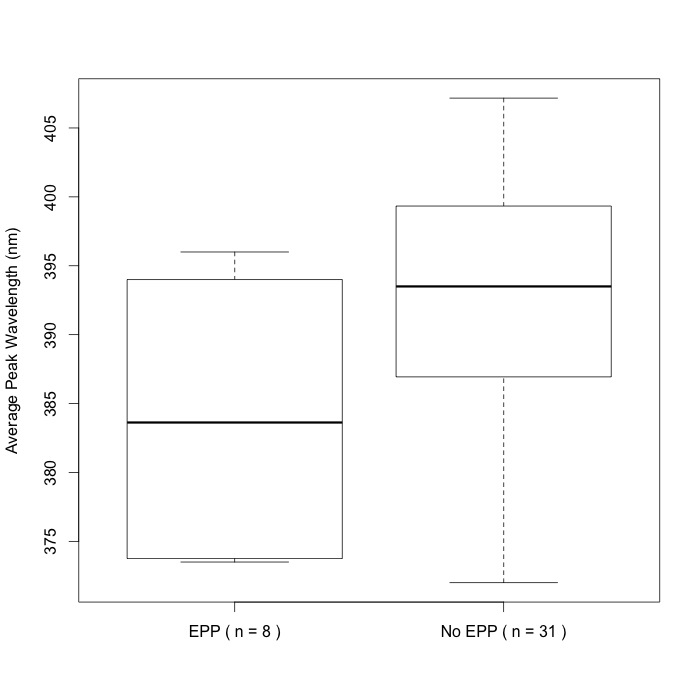

Supplement: Figure S3 — Comparison of feather hue of adult Steller's jays with and without extra-pair parentage (EPP). (TIFF) [file pone.0105257.s003.tiff]

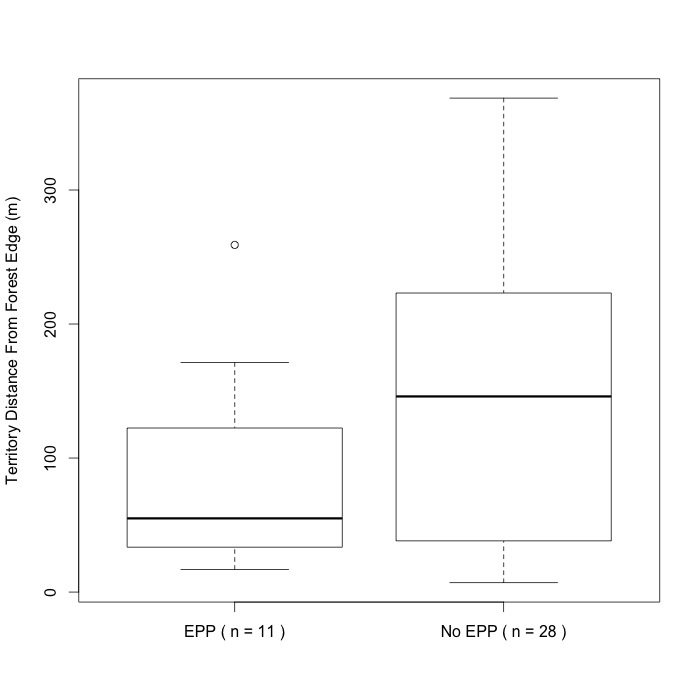

Supplement: Figure S4 — Comparison of nest distance from forest edge (m) of adult Steller's jays with and without extra-pair parentage (EPP). (TIFF) [file pone.0105257.s004.tiff]
